# Specialist Clinicians’ Management of Dependence on Non-Prescription Medicines and Barriers to Treatment Provision: An Exploratory Mixed Methods Study Using Behavioural Theory

**DOI:** 10.3390/pharmacy7010025

**Published:** 2019-03-05

**Authors:** Niamh Fingleton, Eilidh Duncan, Margaret Watson, Catriona Matheson

**Affiliations:** 1 Institute of Applied Health Sciences, University of Aberdeen, Aberdeen AB25 2ZD, UK; 2 Health Services Research Unit, University of Aberdeen, Aberdeen AB25 2ZD, UK; e.duncan@abdn.ac.uk; 3 Department of Pharmacy and Pharmacology, University of Bath, Bath BA2 7AY, UK; m.c.watson@bath.ac.uk; 4 Faculty of Social Sciences, University of Stirling, Stirling FK9 4LA, UK; catriona.matheson@stir.ac.uk

**Keywords:** nonprescription drugs, over-the-counter drugs, drug misuse, substance-related disorders, qualitative research, psychological theory

## Abstract

The aim of the study was to establish how non-prescription medicine (NPM) dependence is treated by doctors in specialist substance misuse treatment services and to identify perceived barriers to providing treatment. An online survey was conducted to establish current practice and whether changes to service provision are needed to facilitate treatment (n = 83). Semi-structured interviews, based on the Theoretical Domains Framework, were conducted to derive a detailed exploration of suggested changes (n = 11). Most survey respondents had encountered cases of NPM dependence. Analgesics containing codeine were the most frequently NPMs of dependence mentioned by respondents. Most respondents were unaware of specific guidelines for the treatment of NPM dependence. The most frequently identified barriers to providing treatment identified by interviewees were limited resources or capacity and the challenges presented by this client group. There was a perception that this client group could be difficult to treat due to comorbidities, and these this client group perceived themselves to be different from people dependent on alcohol or illicit drugs. This study identified a clear need for specific clinical guidelines for the treatment of NPM dependence. Such guidance should be appropriate for specialist and generalist clinicians as the current pressure on resources may force more treatment into general practice. Appropriate care pathways need to be established and defined, and sufficient resources allocated to accommodate this client group.

## 1. Introduction

Non-prescription medicines (NPMs) are medicines which can be obtained and supplied without a prescription. They are also known as over-the-counter (OTC) medicines. NPMs can be obtained from community pharmacies and retail outlets, e.g., supermarkets. They facilitate self-care which brings about benefits for both the individual and the National Health Service (NHS) by reducing the burden of demand on other healthcare settings [1]. Within the UK, Government policy has encouraged self-care [2,3,4,5], including the use of NPMs. The public perceives NPMs to be safer than prescription medicines [6] and associated with low risk [7], but their use can lead to harm and dependence [8]. It is estimated that 2% of the UK adult population has experienced dependence on NPMs, with most being dependent on analgesics containing codeine [9]. In the UK, certain medicines containing codeine can be obtained without a prescription; these come in the form of a syrup (linctus) to treat dry coughs, or a compound analgesic containing codeine (up to 25.6 mg per recommended dose) with paracetamol, ibuprofen or aspirin.

Research into interventions and support for NPM dependence typically revolves around the pharmacy profession, e.g., pharmacy strategies to minimise harm associated with NPM misuse, abuse or dependence [8,10,11,12], and the development of models to identify and treat individuals suspected of inappropriate NPM use [13,14]. To date, there has been no exploration of how NPM dependence is managed by healthcare professionals despite evidence to suggest that services do not reflect the needs of individuals with NPM dependency [15,16]. This study was conducted to explore how NPM dependence is currently managed by doctors within specialist substance misuse treatment services and to identify perceived barriers and enablers to treating NPM dependence.

Behavioural theories have long been used to understand, predict or generate behaviour change in individuals. The capability, opportunity, motivation and behaviour (COM-B) model of behaviour and the linked Theoretical Domains Framework (TDF) have been used to explore perceived influences on behaviour. The COM-B model enables understanding of influences on behaviour [17]. Capability involves physical and psychological components; opportunity involves physical and social factors; and motivations may be either reflective or automatic.

The TDF [18] provides a more detailed understanding of the components of the COM-B model. The TDF is an integrated theoretical framework containing 14 domains synthesised from 33 theories and 128 theoretical constructs, and was developed to investigate determinants of behaviour. It is used to understand behaviour-change processes, design interventions to enhance healthcare practice and assess implementation problems [18].

Developing a literature-based topic survey and topic guide would be difficult due to the sparse literature in this area. Furthermore, research comparing TDF-based interviews and literature-based interviews found that TDF-based interviews may prompt respondents to identify barriers that they would not otherwise report [19]. Thus, the TDF provides a comprehensive framework to prompt the identification of factors that influence behaviour, and consequently potential mediators of behaviour change. In situations where it is not feasible to assess all 14 of the TDF domains, the COM-B model can be used as a screening tool to give an indication of which domains of the TDF to select in conducting more detailed interviews [20].

## 2. Materials and Methods

A mixed methods approach was used incorporating an online survey and semi-structured qualitative telephone interviews.

The target population was doctors in UK specialist substance misuse treatment services. The actual population size could not be easily determined as no current list of clinicians exists. Initially it was hoped that the research team would be able to contact the target group via the Faculty of Addictions at the Royal College of Psychiatrist; however, this was not possible due to data protection regulations and therefore alternative methods had to be used. As such, potentially eligible participants were sent an invitation to participate via the online mailing lists of the Society for the Study of Addiction and the Scottish Addiction Specialist Committee and via the DrugScope (www.dsdaily.org.uk) daily email newsletter. R&D offices were contacted individually to ask if they could provide the authors with contact details of eligible staff within their NHS area or to forward an invitation containing the survey link to eligible staff. This method was used to capture individuals who were not members of the aforementioned organisations. Third sector organisations, Action on Addiction and Turning Point, forwarded an email and survey link to relevant staff while Addaction advertised the survey via their intranet.

Individuals who participated in the online survey and indicated that they were interested in participating in further research were contacted by email and invited to participate in an interview. Only one clinician per NHS administrative area was contacted for interview.

The survey was undertaken with a self-report questionnaire using an online survey platform (www.surveygizmo.com). The questionnaire was developed by the research team with input from a Consultant Psychiatrist in substance misuse and a GP with a special interest in substance misuse. There were screening questions at the beginning of the questionnaire to ensure that respondents were qualified doctors and currently employed to provide drug misuse treatment in the UK within a substance misuse treatment service; those who indicated otherwise were automatically disqualified. A selection of survey questions was derived from the COM-B model to determine which target behaviours needed to change to facilitate the treatment of NPM dependence [21]; questions relating to the COM-B elements of physical skills, strength, limitations and stamina, were excluded because they were not applicable to the target behaviour. Similarly, two statements from both the opportunity and motivation domains were excluded as they were irrelevant (e.g., needing to be reminded to provide treatment for NPM dependence was not applicable as patient presentation cues treatment provision). The survey was available online from May to October 2015. Survey data were exported to SPSS (version 23) for analysis. Descriptive statistics were used to summarise the data. Free-text responses were analysed using content analysis.

The semi-structured interview topic guide was developed based on the TDF [22]. Interview questions were informed by previous studies using the TDF [23,24,25,26,27,28,29]. Interviews were conducted from July to October 2015. The first two interviews were treated as pilot interviews to enhance the clarity of the topic guide; further refinement was deemed unnecessary, and they were included in the analysis. It was intended to conduct interviews until data saturation [30].

Interviews were conducted by telephone and audio-recorded with consent. Audio-recordings were transcribed by a research secretary and then reviewed for accuracy. Personal identifiers and demographic information were removed and stored separately to ensure confidentiality.

Interview data were analysed using directed content analysis [31] and QSR NVivo 10. Coding guidelines were developed to provide a description of each TDF domain (i.e., main themes) and to aid the coding process. Specific beliefs (i.e., subthemes) were identified within each domain by grouping similar quotes. A frequency count for each belief (to represent the number of participants who mentioned the belief) was recorded. A list was generated of specific beliefs from utterances coded in each domain. Double-coding was performed on seven of the 11 transcripts by author ED. ED is a Research Fellow and Health Psychologist with expertise in the theoretical framework and analysis approach used. Additional participant utterances which were of relevance were coded as emergent themes. All TDF domains were explored; however, only those relating to COM-B components to which at least half of survey respondents agreed were targets for change, are reported here.

Ethical approval was obtained from the University of Aberdeen College Ethics Review Board (reference: CERB/2014/10/1130). NHS R&D approval was obtained from all relevant NHS areas.

## 3. Results

### 3.1. Particpants

Questionnaires were completed by 83 (78%) of the 106 individuals who indicated that they were eligible. The data concerning respondents’ demographics and employment characteristics were reviewed and there was no evidence to indicate multiple responses from the same respondent. Most respondents were psychiatrists employed by the NHS and based in England (Table 1). Thirty-six clinicians indicated a willingness to be interviewed. Telephone interviews were conducted with 11 clinicians. Five clinicians were not contacted because a clinician from the same NHS Trust had already participated. The remainder (n = 20) did not respond to attempts to arrange an interview. Interview duration ranged from 22 to 60 min (mean = 33 min). Recruitment stopped when no further interviewees could be recruited within an acceptable timeframe. Data saturation may not have been achieved as new subthemes were identified in the final three interviews [30].

### 3.2. Treatment of NPM Dependence and NPMs of Dependence

Most survey respondents were unaware of specific guidelines for the treatment of NPM dependence (Table 2). The majority of respondents had at least one client who had been dependent on an NPM in conjunction with another substance. Most respondents had a client who had been dependent solely on an NPM (n = 68), and when asked what services they had personally provided for these clients, opiate replacement therapy was most frequently mentioned (n = 56).

Analgesics containing codeine, an opiate, were the most frequently NPMs of dependence in the past 12 months, followed by sleep aids, non-codeine analgesics and smoking cessation products (Table 2). “Other” NPMs specified by survey respondents were: opioid-containing cough medicine (n = 4), cough medicine (unspecified) (n = 3), and an antihistamine (n = 1).

Some interviewees explained that many cases of NPM dependence occur in people receiving prescribed opioids who are either “topping up”, or using non-prescription codeine analgesics once their opioid prescription ended.

### 3.3. Opinions about Treating NPM Dependence

Most survey respondents agreed that substance misuse treatment services (n = 67), GPs (n = 51), and pharmacists (n = 36) should provide treatment for NPM dependence. A greater proportion believed that substance misuse treatment services were better equipped than GPs to provide treatment (n = 50) than vice versa (n = 9) (Table 3). The majority of respondents agreed that the consequences of NPM dependence could be as severe as those of illicit drug dependence (n = 67). Almost half (n = 40) of respondents believed that people with NPM dependence were a different client group compared with those dependent on illicit drugs.

### 3.4. Barriers and Facilitators to Providing Treatment for NPM Dependence

All 14 TDF domains were coded as influences on providing treatment for NPM dependence. A total of 67 specific beliefs were identified (see Supplementary Materials). The survey indicated that there were only two COM-B components to which at least half of participants agreed were targets for change: capability (psychological) and opportunity (physical) (Table 4); therefore, only the TDF domains which map onto these COM-B components (i.e., knowledge; skills; memory, attention and decision processes; behavioural regulation; and environmental context and resources) are presented here. The survey results indicated that motivation was not a target for change (Table 4).

### 3.5. Capability

Over half of survey respondents agreed that they needed to know more about how to provide treatment for NPM dependence (n = 42) (Table 4), indicating that psychological capability was a target for change. The TDF domains pertaining to psychological capability were explored in the interviews and are described in the sections that follow.

#### 3.5.1. Knowledge

This domain includes procedural knowledge and the knowledge and awareness required to provide treatment for NPM dependence. All interviewees demonstrated knowledge of possible treatment options for NPM dependence, including medical treatments (e.g., methadone, buprenorphine) and psychological interventions. This knowledge facilitated the provision of treatment.

“Provide some degree of psychosocial intervention for people, to get an assessment of the level of dependence, the nature of dependence […] it wouldn’t be uncommon for somebody like this to be offered an appointment with one of our medical team relatively early, where any discussion about possible pharmacological intervention might be offered.” (11)

It was not uncommon for interviewees to explain that clients may use NPMs to manage other health problems (e.g., pain, mental health, sleep difficulties), or have physical or psychiatric comorbidities in addition to their NPM dependence. Awareness of the potential for these additional problems, and knowledge of how to assess and manage them, was deemed to be essential by some interviewees.

“There is the need to be also aware to screen routinely and be aware of any sort of co-morbid psychiatric problems, which are fuelling and being masked by the non-prescription drug dependence.” (02)

A few interviewees highlighted the importance of recognising NPM dependence, acknowledging that consequences of NPM dependence could be as severe as those of alcohol or illicit drug dependence, and being aware of the specific risks associated with NPM dependence.

“You have to have an awareness of the specific risks of OTC medicines, I’m thinking particularly of paracetamol and ibuprofen, which come in combined preparations.” (03)

Finally, a couple of interviewees mentioned that they initially had difficulties “finding out what kind of doses people needed” and obtaining information on dose conversions for treatment with methadone or buprenorphine. This information was considered essential in order to provide safe treatment for NPM dependence.

#### 3.5.2. Skills

The skills domain refers to the competencies and abilities required to provide treatment for NPM dependence. Generic addiction skills, and communication and interpersonal skills, were mentioned by almost all interviewees as facilitating treatment. Many skills used in the treatment of alcohol or heroin dependence were viewed as being transferable to treating NPM dependence.

“It’s just standard addiction work, education about the harmful effects of these medications, exploring why they’re using them, […] coping with cravings, looking at people, places and things, other alternatives that they can do, and pointing out the benefits of reduction, we might prescribe something.” (08)

Many interviewees believed communication and interpersonal skills were particularly important for treating NPM dependence. Some interviewees felt that clients dependent on NPMs perceived themselves to be different from people dependent on alcohol or illicit drugs, and consequently different communication and interpersonal skills were needed to engage with these clients and reduce the stigmatisation they may feel.

“Engaging people in a conversation that doesn’t make them feel sort of stigmatised around perhaps having a problem of controlling their OTC drug use.” (02)

All but one interviewee mentioned the various challenges presented by this client group. There was a perception that this client group could be difficult to treat due to physical or psychiatric comorbidities.

“They are a very heterogeneous group, with often physical co-morbidity and psychiatric co-morbidity and sometimes pain issues, so a fairly complex group, with maybe different needs.” (08)

The ability to conduct assessments of both mental and physical health was considered essential. In terms of physical health, the ability to assess pain was the most frequently mentioned, followed by the potential harms from excess consumption of paracetamol or ibuprofen.

“It’s often helpful [to] have people who understand something about physical and mental health issues, because you’ve got to understand whether there are underlying factors going on, so that needs to be part of the assessment skills that people would have.” (06)

#### 3.5.3. Memory, Attention and Decision Processes

Memory, attention and decision processes are the processes involved, and the factors taken into account, when making decisions about providing treatment. All interviewees explained how client circumstances and clinical factors influenced how they provided treatment for NPM dependence, e.g., the NPM a client is dependent on, the dose, reasons for use and for seeking treatment, comorbidities and whether they have tried any previous treatment.

“In making a treatment decision you have a holistic approach, so you look at the whole set up in terms of the patient’s substance use, their environment, their support, their physical and mental health.” (03)

Many interviewees described how their previous experiences influenced how they provide treatment for NPM dependence. These were typically due to experiences that interviewees had with this client group and often involved “learning as you go along”; however, one interviewee referred to a specific incident which influenced practice.

“We had the experience in our service of someone, […] that person ended up dying, and so we just made a policy then, […] where you’re not sure of levels of dependence to go for buprenorphine.” (09)

Interviewees were evenly split on whether providing treatment for NPM dependence was something they knew how to do automatically or something they required time to think about. Those who did not require much time to think about it stated that this was because it was similar to treating heroin dependence, or due to their experience with NPM cases.

“Because I’ve got quite a lot of experience in it I suppose I would know how to treat it automatically.” (04)

Others acknowledged that while the basics of treating NPM dependence “remain the basics of any addiction treatment”, they needed time to think about what medical treatments were appropriate, dose conversions, treatment plans and “the extra dimensions of someone not accepting that they have an addiction”.

“Where people are dealing sometimes with things which are a bit more unusual, you might have to take a bit more time to help you come up with the plan.” (06)

#### 3.5.4. Behavioural Regulation

Behavioural regulation refers to ways of doing things, at an individual or environmental level, that relate to pursuing and achieving desired goals, i.e., providing treatment for NPM dependence. Over half of interviewees said that their service had either developed or was in the process of developing pathways or policies for treating NPM dependence.

“[We’ve written] a couple of flow charts that we use in terms of how we respond to it and so we have a sort of hierarchy of responses.” (04)

Some interviewees described how they had sought literature and guidance to inform their treatment for NPM dependence.

“[We] reviewed some of the literature in terms of what was the evidence, what kind of treatment worked.” (01)

Guidelines were mentioned by some interviewees who believed these might be beneficial for themselves and other treatment providers.

“There are other psychiatrists who need to be reassured about this treatment, and if we have guidelines it will make people easily follow, and treat properly, because I think some psychiatrists, if they don’t have guidelines, or if they don’t have enough information, they will hesitate to treat those people.” (05)

A couple of interviewees said they had attended training or “try to go to [continuing professional development] events” while another described how monitoring outcomes for this client group had changed their practice.

One interviewee suggested that appropriate commissioning of services would facilitate treatment for this client group.

### 3.6. Opportunity

The majority of survey respondents agreed that the following elements were needed to provide treatment for NPM dependence: knowledge about how to do it (n = 42); better materials e.g., guidelines (n = 42), more funds (n = 41) and more time (n = 40), thereby indicating that physical opportunity was a target for change (Table 4). The TDF domains pertaining to physical opportunity were explored in the interviews and are described in the sections that follow.

#### Environmental Context and Resources

This domain refers to any circumstance relating to a clinician’s situation or environment that encourages or discourages treatment provision (including people and organisational factors, e.g., resources, client circumstances).

Almost all interviewees discussed capacity or resource problems. Interviewees described how budget cuts and limited finances resulted in lengthy waiting times for clients and a lack of staff and resources to develop services.

“Addiction psychiatry is suffering a lot now in the NHS, when they want to cut, the first services they cut is the addiction psychiatry, […] so our budget is less than before, it’s smaller and smaller.” (05)

“One of the difficulties is opioid substitution therapy in [region] isn’t that well-resourced and our workers are at capacity, […] if we were to take on this group and look at more rapidly putting them on the opioid substitution therapy, we would very quickly become overwhelmed, and that’s part of our reluctance to do it.” (08)

There was concern that services may be overwhelmed if the numbers of clients were to increase substantially and that services for these clients would be the “first off the list” if funding was to be reduced.

“If we were to start advertising in chemists and things like that, that would be a different ball game altogether, and we’d struggle.” (09)

For one interviewee, the imminent closure of their service was going to prevent them from providing treatment. Despite resource limitations, some interviewees felt they had sufficient time to provide treatment.

“Recently I’ve been lucky because the substance misuse service I work in is not overloaded, so I have time.” (02)

Interviewees described how these clients were often reluctant to attend specialist services or to engage with treatment because they did not see themselves as “drug addicts”. It was acknowledged that specialist services were unlikely to appeal to this client group because “they are mainly suited for people who are using other drugs, so they may feel a bit out of place there”. This made it more difficult for clinicians to provide treatment.

“People who are dependent, are using OTC medications, don’t see themselves as junkies, don’t want to come and sit in the waiting room with homeless, injecting, chaotic people, see themselves as a cut above, so there are difficulties in that area.” (03)

Despite these difficulties, and seemingly in contrast to the problems mentioned above, some clinicians felt that these clients were sometimes easier to treat, or likely to have more successful outcomes, than individuals dependent on illicit drugs. There was a belief that they could be more “compliant” with treatment and had more “recovery capital” than other client groups which made providing treatment for them easier.

“The OTC clients don’t tend to have the other level of chaos and entrenchment that I usually see with class ‘A’ dependent drug users.” (02)

Commissioning arrangements varied between clinicians. One interviewee provided advice and support to GPs to manage NPM dependence but was unable to provide treatment themselves due to commissioning arrangements. Most interviewees were not specifically commissioned to provide treatment for NPM dependence but that they were commissioned to provide treatment for drug or opiate dependence and NPMs fell under that category; it was “not excluded”; or commissioners encouraged it.

“We’re doing it kind of in the remit of opiate dependence, so we’re an opiate-funded service, so if someone’s dependent on an opiate OTC painkiller then you know, we’re able to kind of wangle it that way, but there isn’t any overt or any ring-fenced funding for OTC medication, so we’re really using the funding that came from the HIV crisis.” (07)

A couple of interviewees said that providing treatment for NPM dependence was now specifically within their remit.

“The more recent [service agreements] have included prescription-only medication and OTC.” (01)

Most interviewees indicated that GPs often needed assistance or support from them in order to provide treatment for NPM dependence, or were unwilling to provide treatment at all, due to their lack of knowledge or skills; this encouraged interviewees to either advise GPs on how to treat NPM dependence or to provide treatment for this client group themselves.

“It comes to the attention of the GP, and the GP says, ‘Oh, please help, this lady is buying Nurofen Plus, and she is asking me for help, what should I do?’” (05)

Similarly, some clinicians argued that there was nowhere else from where these clients could receive treatment which encouraged them to provide treatment.

“[We] made a sort of joint decision that we would [provide treatment], as it seemed there wasn’t really anyone else who would.” (04)

Some clinicians admitted they had difficulty providing treatment for NPM dependence due to limited evidence, guidance and literature to refer to, with some attempting to make use of the little they could find.

“It’s not that easy to get any clear guidance of what to do, to be honest, […] there’s not a lot out there, […] there wasn’t loads of [evidence], and it was mostly American, […] in the absence of clear guidelines in this country, it was better than nothing.” (01)

## 4. Discussion

### 4.1. Summary of Main Findings

This study is the first to establish how NPM dependence is managed by doctors within specialist substance misuse treatment services in the UK and to identify the perceived barriers and facilitators to providing treatment for NPM dependence. Most clinicians had encountered cases of NPM dependence although the numbers of clients per respondent were generally low. Most clinicians had prescribed opiate replacement therapy to treat NPM dependence. The most frequently identified barriers to providing treatment were limited resources or capacity. Using theory to identify factors that influence behaviour enables future work to link to the evidence base for developing behaviour change interventions [32,33].

### 4.2. Strengths and Limitations

This study used theory and mixed methods. The interview data was designed to complement and provide depth to the survey findings. The survey was conducted with a national sample of specialist substance misuse treatment providers that included providers from all sectors across the UK. It is the only survey investigating the treatment of NPM dependence by substance misuse treatment providers in the UK to date.

It was not possible to identify, or determine the size of, the target population due to the lack of a current list of substance misuse services in the UK. Attempting to compile such a list would be resource intensive, and such a list would have limited currency due to frequent changes in commissioning arrangements [34]. A range of sampling methods were used to maximise the level of exposure to the target population but this meant that calculating the denominator, and therefore the response rate, was not possible. A good completion rate (78%) was achieved by those indicating that they were eligible to complete the questionnaire (n = 106); however, it is likely that the number of responses was low [35]. This may impact upon the generalisability of the findings.

This research involved doctors in specialist services. It is possible that individuals who present for treatment in general practice may differ from those who present to specialist services, e.g., in terms of severity of dependence. The majority of respondents and interviewees were employed by the NHS; however, third sector organisations are increasingly being commissioned to provide substance misuse treatment services, and therefore respondents may not be representative of the wider population of treatment providers.

### 4.3. Evidence and Guidelines for NPM Dependence

Most survey respondents were unaware of any guidelines regarding the treatment of NPM dependence. While buprenorphine and methadone are indicated for the treatment of illicit opioid dependence, no UK guidelines exist specifically for the treatment of prescription or non-prescription opioid dependence [36]. Participants wanted more knowledge and evidence to inform their practice. Research into the effectiveness of treatment options for non-prescription codeine dependence is warranted so that clinicians can provide evidence-based treatment and have confidence in treatment decisions. Information regarding dose conversions (i.e., how much buprenorphine or methadone to prescribe based on the amount of codeine that the individual was consuming) was also considered essential to provide safe medical treatment. If specialist clinicians who provide treatment for substance dependence on a daily basis experience difficulty due to the lack of evidence or guidance, it is almost certain that GPs will experience these problems to a greater extent.

### 4.4. Commissioning, Resources and Capacity

Commissioning arrangements prevented one clinician from directly providing treatment for NPM dependence. This situation is not unique. According to a 2011 report on the configuration and commissioning of treatment services in England, 46% were funded or commissioned to meet the needs of people dependent on prescription and/or NPMs, 16% were not funded but could target and monitor them, 31% were not funded but could work with them, and 6% were not funded and could not work with them [34].

Almost all participants identified limited resources or capacity as potential barriers to providing treatment. Some services were already experiencing limited capacity and resources, and reported lengthy waiting times for appointments. Some believed that if awareness of treatment availability increased, specialist services might struggle to cope with demand. These beliefs are not unfounded. In 2011, local drug and alcohol partnerships throughout England expressed concerns about how increased demand for treatment for prescription or NPM dependence could be met within the current resources [37]. Financial circumstances have since worsened. A survey of 189 drug and alcohol treatment services from across England found that 71 services reported a reduction in funding in 2014 with an overall reduction in funding of around 16.5% [34]. Treatment in primary care or a shared-care approach may be necessary to address limited resources.

### 4.5. Application of the TDF

There is increasing awareness that interventions to change behaviour should draw on theories of behaviour and behaviour change in their development [38]. The use of theory can provide explanations regarding how or why behaviours change and offer an approach to intervention development [39]. Theory-based interventions use an explicit causal pathway and allow those developing the intervention to avoid implicit causal assumptions which may lack evidence [39]. The TDF provides a comprehensive framework to prompt the identification of factors that influence behaviour, and consequently identify potential mediators of behaviour change. The findings of this research indicated that psychological capability and physical opportunity were targets for change. This enables the identification of appropriate intervention functions to facilitate the treatment of NPM dependence and policy options to support implementation. Literature indicates that interventions which involve education, training and enablement may be appropriate for addressing problems around psychological capability [17]; therefore, the development of guidelines, as suggested by interviewees, is likely to be an appropriate strategy to address issues around psychological capability. Research indicates that interventions involving restriction, environmental restructuring and enablement may be appropriate for issues pertaining to physical opportunity [17]; consequently, establishing and defining care pathways and allocating of resources to accommodate this client group is likely to be an appropriate strategy to alleviate problems regarding physical opportunity.

## 5. Conclusions

Most respondents had encountered cases of NPM dependence. This study identified a clear need for specific clinical guidelines for the treatment of NPM dependence; research into effective treatment methods for this client group may be required to ensure that guidelines are evidence-based. Treatment guidelines should be appropriate for specialist and generalist clinicians as the current pressure on resources may force more treatment into general practice. Limited resources and capacity were identified as potential barriers to providing treatment. Appropriate care pathways need to be established and defined with sufficient resources allocated to accommodate this client group to ensure their needs are met. The use of a theoretical framework allows future work to link to the evidence base for developing behaviour change interventions.

## Figures and Tables

**Table 1:** Participant and service characteristics.

	Survey		Interviews	
	%	(n)	%	(n)
**Sex**	(N = 78)		(N = 11)	
Male	66.7	(52)	63.3	(7)
Female	33.3	(26)	36.4	(4)
**Age (years)**	(N = 77)			
Range	33–70		34–58	
Median (IQR)	47 (40–52)		49 (44–54)	
**Type of doctor**	(N = 83)			
Psychiatrist	80.7	(67)	81.8	(9)
General Practitioner	18.1	(15)	18.2	(2)
Other qualified doctor	1.2	(1)	0.0	(0)
**Duration working as specified type of doctor (in years)**	(N = 77)			
Range	5–40		8–32	
Median (IQR)	19 (12–25)		20 (15–27)	
**Duration working in addiction or substance misuse (in years)**	(N = 77)			
Range	1–35		1–26	
Median (IQR)	10 (6–16.5)		12 (8–18)	
**Country**	(N = 83)			
England	69.9	(58)	81.8	(9)
Northern Ireland	2.4	(2)	9.1	(1)
Scotland	21.7	(18)	9.1	(1)
Wales	6.0	(5)	0.0	(0)
**Sector**	(N = 83)			
National Health Service	88.0	(73)	72.7	(8)
Third sector	8.4	(7)	18.2	(2)
Private	3.6	(3)	9.1	(1)
**Are the services targeted specifically at any of the following groups?** ^1^	(N = 83)			
Not targeted at any specific group	78.3	(65)	90.1	(10)
Homeless	14.5	(12)	9.1	(1)
Offenders	13.3	(11)	9.1	(1)
Women	13.3	(11)	9.1	(1)
Ethnic minorities	12.0	(10)	9.1	(1)
Young people	4.8	(4)	9.1	(0)
Other	7.2	(6)	0.0	(0)
**How are clients referred to the service?** ^1^	(N = 83)			
By GP or other health professional	86.7	(72)	90.1	(10)
Self-referral	80.7	(67)	81.8	(9)
Other	50.6	(42)	45.5	(5)
**What services do you personally provide for clients who misuse drugs?** ^1^	(N = 83)			
Managed reduction plan	91.6	(76)	100.0	(11)
Prescribing of opiate replacement therapy	90.4	(75)	100.0	(11)
Referral to other health services	86.7	(72)	100.0	(11)
Outpatient detoxification	84.3	(70)	100.0	(11)
Assessment and pre-treatment services	79.5	(66)	72.7	(8)
Therapeutic approaches	68.7	(57)	81.8	(9)
Referral to other social or support services	68.7	(57)	9.1	(1)
Inpatient detoxification	51.8	(43)	72.7	(8)

Note: ^1^ Percentages total more than 100 as multiple response options were allowed. Not all 83 respondents provided valid answers. Actual numbers of respondents are shown next to the individual question in brackets. Results show valid percentages.

**Table 2:** Participant and service characteristics.

	Survey		Interviews	
	%	(n)	%	(n)
**Are you aware of any guidelines for the treatment of NPM dependence?**	(N = 83)		(N = 11)	
Yes	31.3	(26)	54.5	(6)
No	68.7	(57)	45.5	(5)
**Have you ever had a client who has been dependent on an NPM, in conjunction with dependence on illicit drugs, alcohol or prescribed medicines?**	(N = 83)			
Yes	88.0	(73)	100.0	(11)
No	12.0	(10)	0.0	(0)
**Estimated number of these clients:**				
**Currently**	(N = 61)			
Median (IQR)	2 (1–5)		2 (1–5)	
**Within the past 12 months**	(N = 69)			
Median (IQR)	5 (2–10)		5 (1–20)	
**Have you ever had a client who has been dependent solely on NPMs?**	(N = 83)			
Yes	81.9	(68)	90.1	(10)
No	18.1	(15)	9.1	(1)
**Estimated number of these clients:**				
**Currently**	(N = 60)		(N = 10)	
Range	0–20		0–10	
Median (IQR)	2 (1–4)		2 (0.75–4.25)	
**Within the past 12 months**	(N = 64)		(N = 10)	
Range	0–25		0–20	
Median (IQR)	4 (2–10)		6 (0.75–12.5)	
**What services have you personally provided for clients who have been solely dependent on NPMs?**	(N = 68)		(N = 10)	
Prescribing of opiate replacement therapy	82.4	(56)	80	(8)
Assessment and pre-treatment services	77.9	(53)	60	(6)
Managed reduction plan	72.1	(49)	70	(7)
Therapeutic approaches	63.2	(43)	40	(4)
Outpatient detoxification	54.4	(37)	70	(7)
Referral to other health services	51.5	(35)	60	(6)
Referral to other social or support services	26.5	(18)	20	(2)
Inpatient detoxification	8.8	(6)	30	(3)
**Of clients solely dependent on NPMs within the past 12 months, how many have been dependent on the following?** ^1^				
**Analgesics containing codeine**				
Range	0–30		0–30	
Median (IQR)	3 (2–6)		4.5 (0–14.75)	
0	4		3	
1 or more	58		7	
**Non-codeine containing analgesics**				
Range	0–5		0–5	
Median (IQR)	0 (0–0.25)		0 (0–2.75)	
0	35		7	
1 or more	11		3	
**Sleep aids (non-herbal)**				
Range	0–5		0–5	
Median (IQR)	0 (0–1)		0 (0–1.25)	
0	33		7	
1 or more	13		3	
**Smoking cessation products**				
Range	0–2		0–0	
Median (IQR)	0 (0–0)		0 (0–0)	
0	40		0	
1 or more	1		0	

Note: ^1^ Respondents indicating they ever had a client dependent solely on an NPM were asked this question and told to enter “0” if none. Many did not answer the question as instructed and consequently it was not possible to differentiate between those who had “0” clients and missing responses. No percentages or N are provided as the denominator is unknown. Those who provided any number, including 0, were used to calculate the median and IQR.

**Table 3:** Opinions about treating non-prescription medicine dependence.

	Strongly Disagree or Disagree		Neutral		Agree or Strongly Agree	
Statement (n = 83)	%	(n)	%	(n)	%	(n)
I would feel more comfortable treating illicit drug dependence than OTC medicine dependence.	**39.8**	**(33)**	31.3	(26)	28.9	(24)
I would feel more comfortable treating OTC medicine dependence than illicit drug dependence.	**57.8**	**(48)**	36.1	(30)	6.0	(5)
It would be just as challenging to treat OTC medicine dependence as it would be to treat illicit drug dependence.	13.3	(11)	18.1	(15)	**68.7**	**(57)**
Substance misuse treatment services should provide treatment for OTC medicine dependence.	6.0	(5)	13.3	(11)	**80.7**	**(67)**
GPs should provide treatment for OTC medicine dependence.	20.5	(17)	18.1	(15)	**61.4**	**(51)**
Pharmacists should provide treatment for OTC medicine dependence.	32.5	(27)	24.1	(20)	**43.4**	**(36)**
Substance misuse treatment services are better equipped to treat OTC medicine dependence than GPs.	15.7	(13)	24.1	(20)	**60.2**	**(50)**
GPs are better equipped to treat OTC medicine dependence than substance misuse treatment services.	**63.9**	**(53)**	25.3	(21)	10.8	(9)
The consequences of OTC medicine dependence can be as severe as those of illicit drug dependence.	14.5	(12)	4.8	(4)	**80.7**	**(67)**
People with OTC medicine dependence are a different client group than those with illicit drug dependence.	20.5	(17)	31.3	(26)	**48.2**	**(40)**

Note: GPs: General Practitioners; OTC: over-the-counter. The most frequently indicated responses are presented in bold.

**Table 4:** Factors influencing the provision of treatment for non-prescription medicine dependence.

Imagine a Client Solely Dependent on an OTC Medicine Presented to you for Treatment within the next 12 Months. Please Indicate the Extent to Which you Agree or Disagree that you Would Need Each of the Following Things in Order to Provide them with Treatment. To Provide Treatment for the Client, I would have to… (n = 80)	Strongly Disagree or Disagree		Neutral		Agree or Strongly Agree	
	%	(n)	%	(n)	%	(n)
Know more about why it was important e.g., have a better understanding of the benefits of providing treatment for OTC medicine dependence. ^1^	**47.5**	**(38)**	20.0	(16)	32.5	(26)
Know more about how to do it e.g., have a better understanding of effective ways to treat OTC medicine dependence. ^1^	31.3	(25)	16.3	(13)	**52.5**	**(42)**
Have better mental skills e.g., develop reasoning, logic, comprehension. ^1^	**53.8**	**(43)**	31.3	(25)	15.0	(12)
Have more mental strength. ^1^	**55.0**	**(44)**	32.5	(26)	12.5	(10)
Overcome mental obstacles. ^1^	**51.3**	**(41)**	31.3	(25)	17.5	(14)
Have better mental stamina e.g., develop greater capacity to maintain mental effort. ^1^	**53.8**	**(43)**	32.5	(26)	12.5	(10)
Have more time to do it e.g., create sufficient time to address the issue during consultations. ^2^	27.5	(22)	22.5	(18)	**50.0**	**(40)**
Have more funds to support the provision of treatment. ^2^	23.8	(19)	25.0	(20)	**51.3**	**(41)**
Have better materials e.g., acquire guidelines for the task. ^2^	27.5	(22)	20.0	(16)	**52.5**	**(42)**
Have more people around me doing it e.g., feel that there are other people around me providing treatment for OTC medicine dependence. ^2^	**43.8**	**(35)**	25.0	(20)	31.3	(25)
Have more support from others e.g., have my colleagues behind me. ^2^	37.5	(30)	22.5	(18)	**38.8**	**(31)**
Feel that I want to do it enough e.g., feel more of a sense of satisfaction from doing it. ^3^	**45.0**	**(36)**	33.8	(27)	20.0	(16)
Feel there is enough of a need to do it e.g., care more about the negative consequences of not doing it. ^3^	**40.0**	**(32)**	23.8	(19)	36.3	(29)
Believe that it would be a good thing to do e.g., have a stronger sense that I should do it. ^3^	**37.5**	**(30)**	36.3	(29)	26.3	(21)

Note: OTC: over-the-counter. ^1^ From “capability” domain of COM-B model; ^2^ from “opportunity” domain of COM-B model; ^3^ from “motivation” domain of COM-B model. The most frequently indicated responses are presented in bold.

## Supplementary Materials

The following are available online at https://www.mdpi.com/2226-4787/7/1/25/s1, Table S1: Summary of belief statements and illustrative quotes assigned to the theoretical domains.
