# Supplementary material for: Specialist Clinicians’ Management of Dependence on Non-Prescription Medicines and Barriers to Treatment Provision: An Exploratory Mixed Methods Study Using Behavioural Theory
Source: Pharmacy (Basel). 2019 Mar 5;7(1):25. doi: 10.3390/pharmacy7010025 (PMC6473901; doi:10.3390/pharmacy7010025)
Supplement: Supplementary file 1 [file pharmacy-07-00025-s001.pdf]

Table S1. Summary of belief statements and illustrative quotes assigned to the theoretical domains

**Theme / domain**<br>Subtheme / specific belief	**Illustrative quote**	**Frequency out of 11**
**KNOWLEDGE**		**11**
Knowledge of treatment options	“You need to have an understanding of the medications that are used and its treatment.” (03)	11
Awareness of the potential for other health problems or comorbidities	“We’d always want to make sure that the reasons why people were taking the medication were looked at, so it’s drifting into the whole issue of pain management and pain clinics, we’re not keen to put people onto replacement treatment without being sure if underlying conditions have been addressed really.” (06)	5
Awareness of NPM dependence and/or the importance of treating it	“Realisation that OTC drug dependence could be just as serious as any other illicit drug or alcohol dependence.” (02)	4
Knowledge of dose conversions	“One of the tricky things at first was knowing about dose conversions and what dose of buprenorphine people might need, and obviously it was difficult to get information on that.” (01)	2
**SKILLS**		**11**
Generic addiction skills	“We’re used to treating opiate dependence in the form of heroin users, so we’ve got those, and obviously you can kind of transfer some of those skills, which is what happened, [...] it’s not that much different to treating heroin users, so you use some of those skills.” (01)	10
Communication or interpersonal skills	“I think the added skill with over-the-counter medicines [is that] you’re contending with people sometimes viewing themselves as a bit different than other people, […] I mean part of it is actually making people feel that they are being listened to as an individual, [...] I think some of it can be about helping people to feel that you have got the skills to understand.” (06)	9
Ability to conduct assessments of physical and mental health	“I think adequate assessment is pretty critical, and that is a complicated thing, so you might have to assess the pain, you might have to assess anxiety, or depression.” (04)	5
Ability to tailor the treatment to the individual	“We very much have to individualise treatment plans to suit the patient, I think it’s because they are very heterogeneous group, […] it’s case by case, trying to match the treatment to the patient, because they have very different needs.” (08)	5
**SOCIAL OR PROFESSIONAL ROLE AND IDENTITY**		**11**
Providing treatment for this client group is compatible with my role in addiction	“The way we see over-the-counter dependence and treatment is a part of our routine day-to-day work, because we are a drug treatment service, whether it’s over-the-counter or illicit or any other kind of drug.” (10)	11
We have a duty to provide treatment for this client group	“We have a duty to provide treatment for people who present to our service, we don’t turn people away if it’s appropriate for them to be treated.” (03)	6
We have the expertise to deal with it	“We are the people who are well prepared to deal with the complexity of those people, so anyone who is taking OTC medication, [...] we can help them better in terms of follow-up or management, because that’s our field and we know how could we support them in that way.” (05)	2
Uncertainty over role	“There was a degree of uncertainty as to, I don’t know whether we should be treating them, or GPs.” (06)	2
**BELIEFS ABOUT CAPABILITIES**		**11**
I am fairly confident I can provide treatment for NPM dependence in general	I think we’ve got the skills and experience to deal with those sorts of behavioural type problems, so I’ve got the confidence.” (09)	11
My confidence is relatively low	“I would say my confidence is relatively low.” (11)	3
My confidence depends on the complexity	“If they’ve got very significant co-morbid trauma and mental health difficulties, it may be trying to stabilise their addiction, and doing some mental health work with them, so I’d be less confident with that group, so the more complex physical, psychiatric morbidity they have, the more difficult it is, but as for getting them through an in-patient detox, I’m confident, but I’m less confident about them not relapsing.” (08)	1

Table S1. Summary of belief statements and illustrative quotes assigned to the theoretical domains

**Theme / domain**<br>Subtheme / specific belief	**Illustrative quote**	**Frequency out of 11**
**OPTIMISM**		**11**
This client group is easier to treat, or has better outcomes, than other clients	"I think they have a better overall outcome, […] a better prognosis than our typical opiate dependent patient." (07)	8
I am optimistic about this group and think they can do well	"I think this client group can do well, […] I'd be optimistic enough." (08)	7
I am not particularly optimistic	"At this point in time I would put my optimism at less than 50%, [...] from experience of having worked with OTC preparations, I think that there's more than 50% chance that they will drop out of treatment, and then of sticking to the treatment." (10)	3
I have mixed feelings	"We will treat them, but the outcomes can be hard to predict, and it would depend on the individual patient, some patients you see it's relatively straightforward, some are much more complex and you can see it being a rocky road." (03)	3
**BELIEFS ABOUT CONSEQUENCES**		**11**
Not providing treatment would have negative consequences for clients	"I presume a number of people will change their behaviours, and I imagine some people will go on and develop bigger problems, I guess it depends what they're taking, what physical or mental health issues may come in as part of the sequelae of it, so if we don't treat, you imagine over a whole sample, people will be less well." (06)	10
Providing treatment may result in positive outcomes for clients	"First of all I want to see hopefully an improvement in the medical status, like less gastrointestinal bleed, and people's haemoglobin levels coming up, and less emergency hospital presentations, so that's with my harm reduction head, and then hopefully clients getting control of their dependence, reducing that level of dependence, and that their mental health and overall quality of life is improved as a result of getting more appropriate treatment, whether that's sort of medication like antidepressants, or accessing psychological services." (02)	10
Providing treatment may result in positive outcomes for us	"Well the positive outcome for me is I like working with this client group." (02)<br>"The desirable outcome is that we successfully treat them and can add to our tally of successful outcomes." (03)	8
Providing treatment may result in negative outcomes for clients	"I think there's still the risk of, the patients that I've had, there's been a risk of recurrence, they come back maybe two or three times." (09)	3
Providing treatment may result in negative outcomes for us	"I think the negative thing would be the impact on caseload and the fact that other people are waiting longer." (07)	3
Not providing treatment would have very little impact	"I don't think it would make much difference, because I don't think we're doing very much, so I don't think our service is making much of an impact on OTC dependence." (09)	1
**REINFORCEMENT**		**10**
There are no incentives to provide treatment	"It's not on our key performance indicators for our contract." (09)	5
Seeing these clients do well encourages me to provide treatment	"Having patients that we've been successful with would encourage us to keep going, […] there's satisfaction, I mean clearly you're helping people and that's an incentive to keep on with it." (08)	5
I find these clients interesting or enjoyable to work with	"It's quite interesting trying to get involved in something that was an up-and-coming problem." (01)	4
Providing treatment for these clients improves service figures	"I guess you could see people with OTC dependency as potentially easy wins for the service in terms of payment by results scenarios, because you should get more successful outcomes with them than with street heroin users, so I guess there are some incentives." (02)	3

Table S1. Summary of belief statements and illustrative quotes assigned to the theoretical domains

**Theme / domain**<br>Subtheme / specific belief	**Illustrative quote**	**Frequency out of 11**
**INTENTIONS**		**11**
I intend, or made the decision, to provide treatment for NPM dependence	"Most of the patients have come over to us, we take them on for treatment, with intention of treating them." (10)	9
I try to support GPs to treat these clients instead	"We would typically just be giving advice to the GP in the first instance, unless we thought they were very complicated and in which case we might take them over on the spot, but yes, we'd want the GP to manage it if possible, […] it seemed that wasn't really anyone else who would do that, to support the GPs in doing it, so, so we chose to do that." (04)	2
**GOALS**		**11**
Providing treatment for this client group is important	"They are people who have problems that would benefit from help, so yes, we recognise they're important." (04)	8
These clients make up a small proportion of our overall numbers	"As a percentage of my casework, it's very small." (07)	4
NPM dependence is less of an issue or priority than alcohol or illicit drug use	"It's less of a big issue than illicit drug use in a way, because the adverse consequences of illicit drug use are much broader and deeper." (03)	4
These clients need a service just as much as anyone else	"For each individual client that comes through, I see no reason why they should be of any less priority than anybody else, I think we'd be potentially failing as a service if it was the case." (11)	3
**MEMORY, ATTENTION AND DECISION PROCESSES**		**11**
Client circumstances and clinical factors influence how I treat NPM dependence	"We would do an assessment, and we would try to stabilise their use first and get them engaged in treatment, we'd be doing education with them, and initially harm reduction work, and then identify what the immediate goals were, are they anaemic, are they getting a lot of tummy pain, what are their basic bloods like, is there any sign of liver upset, upsetting the liver function and the kidney function, are there any acute mental health issues that need to addressed just now, and do they need an antidepressant, do they need referral to the mental health team." (08)	11
Previous experience influences how I treat NPM dependence	"Previous experience, having tried different kinds of medication treatment and seen the levels of success and all that." (10)	7
I need time to think about how to treat it	"I would say it does tend to take a bit more thought, the basics of it remain the basics of any addiction treatment, [...] I may deliberately take a bit more time over it, because I don't have a clear, you know, this is kind of tapping into slot B, this is what I need to do." (11)	6
I didn't need to think about it very much	"I just approached it in the same manner as I do normally, […] I sort of see it as being a similar job to dealing with someone that's got a heroin dependence." (09)	5

Table S1. Summary of belief statements and illustrative quotes assigned to the theoretical domains

**Theme / domain**<br>Subtheme / specific belief	**Illustrative quote**	**Frequency out of 11**
**ENVIRONMENTAL CONTEXT AND RESOURCES**		**11**
Limited resources or capacity	"The main problem is withdrawal of finances and cuts in medical staffing or people with the skill mix like myself to be able to manage these clients holistically, all the services in the NHS trust that I work for, have mostly been lost to non-statutory providers, who do not employ many medical staff, and tend not to employ staff like myself, who hold CCT's [Certificate of Completion of Training] in psychiatry or addiction psychiatry, so I think that the care of these individuals is likely to be fragmented and individuals like myself are facing redundancy." (02)<br>"We have so much pressure on our waiting times, so that would be the only thing that would put me off, if we're failing to make our waiting times and this service isn't actually funded, so I think if the numbers do increase significantly that is going to be an issue for the directorate, because our funding is really ring-fenced to treat illicit opiate users, especially injecting use, so if we have pressure and have to tighten our boundaries, then these patients will be the first off the list I think." (07)	10
Challenges presented by this client group	"Some of these patients are very complex, and have got physical and mental health comorbidities." (03)<br>"They don't see themselves as having an addiction quite often, so there's often increased problems, in terms of them accepting that they do need the full range of interventions that we provide someone with addiction, people tend to see them as being slightly different or special, and not wanting to comply with daily dispensing, supervised treatment." (07)<br>"Obviously they could get them over-the-counter, so in a sense, whatever treatment you offer, they are still able legally to get that preparation, so I think that can be another facet in terms of peoples motivation for change." (06)	10
Commissioning arrangements	"There's now specifically a remit for drug treatment services to manage people with prescription and over-the-counter drug dependency." (03)<br>"Not specifically, but you know, we're commissioned to provide treatment to anyone that turns up with a dependence to drugs." (09)<br>Interviewer: "You're unable to provide it yourself, because it's not commissioned?" Clinician: "Yes". (02)	9
GPs are often unable or unwilling to treat it themselves	"Some GPs will do it happily, and then others just wouldn't either have the confidence or would just not really entertain it, and I can understand, if they've not got the resources or the skills, or the training, it's fair enough isn't it?" (01)	8
There is nowhere else for these clients to go	"We are the last place that the patients can seek treatment for whatever the dependency is, that's available in the community, in this Borough, […] if it's not us, then there isn't anywhere else that they can approach." (10)	7
Limited evidence base or guidance	"I think part of the difficulty in working with this client group is there isn't a lot of evidence-base out there to base your practice on." (08)	7
There is demand or increased demand for treatment for NPM dependence	"This is becoming an increasing problem." (05)<br>"The vast majority of my opiate detoxes here in [region] are for over-the-counter medicines, which is probably very different from the mainland, we have very big rates of over-the-counter medication and prescription drug abuse here." (08)	6
These clients are easier to treat than other clients	"They may well have more social capital, some more things going for them than some of the other people what we're seeing." (06)	6
I have sufficient time to provide treatment for these clients	"I think I have long enough to see patients when I see them for assessments or reviews." (03)	5
There is very little demand for treatment	"It's not something that we commonly see, […] the problem is relatively uncommon in terms of presentation to us." (11)	2
Closure of service	"My service shuts at the end of [month], and my confidence that the needs of this group will be met from then on is zero." (02)	1

Table S1. Summary of belief statements and illustrative quotes assigned to the theoretical domains

**Theme / domain**<br>Subtheme / specific belief	**Illustrative quote**	**Frequency out of 11**
**SOCIAL INFLUENCES**		**9**
Clients' views influence me	"Their view is quite important because if they believe in a particular strategy then it's going to be more likely to work, so we would want to discuss that strategy with them quite carefully." (04)	8
Colleagues influence me	"It's not a decision made by the therapist alone, all the patients with complex problems are brought to the multidisciplinary team meeting and they decide on what is the best way of treating that patient." (10)	7
Clients' views do not influence me	"Usually in general we don't let [clients] to influence our treatment, because we know the best evidence." (05)	2
Colleagues do not influence me	"I suppose I'm in the position really where it's a bit up to me, so I would be more in the position of actually trying to not let other people put artificial obstructions." (06)	2
Knowing that others think it is worth treating encourages me provide treatment	"Knowing that some other areas do it, made us think, well we'll start doing it, but I know there's some areas that still don't do it as well, but I think knowing that some addiction services were starting to treat this group, we kind of decided to start doing it." (01)	2
**EMOTION**		**7**
Treating these clients can be stressful or frustrating	"Certainly there are times when I guess a sense of frustration is easier to come to in this particular group, [...] the more I invest in somebody and the more it then doesn't work, then that's likely to cause a kind of subsequent adverse reaction against it." (11)	3
My emotions don't really influence my treatment provision	"I hope there wouldn't be many more laden issues than what I would apply to others." (06)	3
I am concerned about potential consequences of treating these clients	"I'm concerned about them mixing with other individuals who might have maybe more severe problems, so on occasions I've done consultations with the GP and the client in a primary care setting." (02)	2
I am concerned about consequences of not treating these clients	"I obviously have concern for people who are causing harm to themselves, and I wish to help them cease those harmful habits, and improve their health and functioning," (03)	2
It would be sad not to provide treatment for these clients	"If I don't provide that treatment, I think it's really sad." (05)	1
I feel insecure when treating these clients	"You feel very much like you're trying to cobble together for the patient, and you feel a wee bit insecure, because there isn't the evidence base there to guide you." (08)	1

Table S1. Summary of belief statements and illustrative quotes assigned to the theoretical domains

**Theme / domain**<br>Subtheme / specific belief	**Illustrative quote**	**Frequency out of 11**
**BEHAVIOURAL REGULATION**		**10**
Our service has developed or is in the process of developing pathways or policies for treating NPM dependence	"We have established a pathway to assess and treat people who are addicted to prescribed medication, or people who are dependent on over-the-counter medication." (05)	6
I have sought out literature or read guidelines	"What we use is the clinical guidelines for drug treatment that came out in 2007, […] there is, I think, a small section on over-the-counter preparations, so we have to refer to the guidelines when we make decisions on treatment." (10)	4
Guidelines would be helpful	"Guidelines would be very helpful, […] that would be lovely to see, to have, to get some guidance." (08)	4
I attend training events on NPM dependence	"I have been on some [Royal College of General Practitioners] training." (01)	2
I monitor the outcomes for this client group	"I've audited and that was when I realised that this group just weren't coping with the standard detox, [...] I monitor every year, the completion rate of the opiate detoxes on the ward, and you know, over the past five years since I've changed to using codeine, I've become, our completion rate has gone up from 50 to 90 odd percent, so that's good, and I monitor that every year." (08)	1
Proper commissioning of services would help	"If the services were commissioned properly, […] I've also flagged up again, with our commissioners and the clinical commissioning group about the absence of a commissioned care pathway for this service user group." (02)	1
